# Study on the Liquid Transport on the Twisted Profile Filament/Spun Combination Yarn in Knitted Fabric

**DOI:** 10.3390/polym17152065

**Published:** 2025-07-29

**Authors:** Yi Cui, Ruiyun Zhang, Jianyong Yu

**Affiliations:** 1 Key Laboratory of Textile Science and Technology, Ministry of Education, College of Textiles, Donghua University, Shanghai 201620, China; 2150143@mail.dhu.edu.cn; 2 Innovation Center for Textile Science and Technology, Donghua University, Shanghai 201620, China; yujy@dhu.edu.cn

**Keywords:** twisted combination yarn, hydrophobic-hydrophilic gradient, liquid migration, liquid moisture supply, wicking

## Abstract

The excellent moisture transport properties of yarns play a crucial role in improving the liquid moisture transfer behavior within textiles and maintaining their thermal-wet comfort. However, the current research on the moisture management performance of fabrics made from yarns with excellent liquid transport properties primarily compares the wicking results, without considering the varying requirements of testing conditions due to differences in human sweating rates during daily activities. Moreover, the understanding of moisture transport mechanisms in yarns within fabrics under different testing conditions remains insufficient. In this study, two types of twisted combination yarns, composed of hydrophobic profiled polyester filaments and hydrophilic spun yarns to form a hydrophobic-hydrophilic gradient along the axial direction of the yarn, were developed and compared with profiled polyester filaments to understand the liquid migration behaviors in the knitted fabrics formed by these yarns. Results showed that hydrophobic profiled polyester filament yarn demonstrated superior liquid transport performance with infinite saturated liquid supply (vertical wicking test). In contrast, the twisted combination yarns exhibited better moisture diffusion properties under limited liquid droplet supply conditions (droplet diffusion test and moisture management test). These contradictory findings indicated that the amount of liquid moisture supply in testing conditions significantly affected the moisture transport performance of yarns within fabrics. It was revealed that the liquid moisture in the twisted combination yarns migrated through capillary wicking for moisture transfer. Under an infinite saturated liquid supply condition, the higher the content of hydrophilic fibers in the spun yarns, the greater the amount of moisture transferred, demonstrating an excellent liquid transport performance. Under the limited liquid droplet supply conditions, both the volume of liquid water and the moisture absorption capacity of the yarn jointly influence internal moisture migration within the yarn. It provided a theoretical reference for testing the internal moisture wicking performance of fabrics under different states of human sweating.

## 1. Introduction

Clothing, as a substance in direct contact with the human body, regulates the microclimate between the human body and the garment and maintains the dryness and comfort of the human body by transporting perspiration away from the skin surface. The moisture transfer behavior of textiles directly affects wearing comfort, which has been gaining further attention in the area of functional textile moisture management [1,2]. The liquid migration in fabrics contains absorption, wicking, and evaporation processes. Wicking is the spontaneous imbibition of a wetting liquid into a porous medium by capillary action, which can only occur when the liquid wets the capillary spaces between the porous medium [3,4]. Fabrics can be considered as a network constructed by yarns, and moisture transport within fabrics occurs along these yarns, indicating that yarns play an important role in facilitating liquid moisture transfer through the wicking effect within fabrics. Lu et al. [5] investigated the influence of yarn structure on liquid moisture transport behavior and found that the liquid transport was determined to a large extent by the fiber arrangement and structures in yarns. Mhetre [6] carried out wicking experiments on a range of cotton and polyester fabrics and showed that the wicking in fabrics was determined by the wicking behavior of the yarn, the thread spacing, and the yarn migration rate.

As is generally known, hydrophilic fibers can absorb moisture well but have poor moisture transportation and release, whereas hydrophobic fibers show the opposite [7]. The yarns contain hydrophilic and hydrophobic fibers with different proportions and distributions that can take advantage of both fibers and maximize the liquid moisture transport performance. There are three types of moisture transport yarns, including the spun yarns uniformly blended different hydrophilic and hydrophobic fibers [8,9,10], axial non-uniform bicomponent yarn constructed from different hydrophilic and hydrophobic fibers [11], and radial non-uniform structured yarns with different hierarchical cross-sections, such as wrapped yarns, core-spun yarns, and twisted combination yarns, made by different hydrophilic-hydrophobic fibers, yarns, and filaments [12,13,14]. The twisted combination yarns are directly produced using twisting machines without the need for new or modified yarn production equipment, making them more practical during processing. The profiled filament with a large specific surface area and continuous water transport channels can effectively enhance the capillary effect and wicking performance [15]. The excellent moisture absorption of hydrophilic yarns twisted with profiled filament could form alternately spaced hydrophilic/hydrophobic regions that provided power for capillary flow and improved the migration rate of moisture in the yarn along the axial direction to achieve moisture transport performance significantly.

Many studies have been conducted to assess the moisture management properties of fabrics, relying on the array of tests under varying conditions [16]. The human body’s local non-perceptible perspiration rate ranges from 0.02 to 0.07 mg/(cm^2^∙min), while perceptible perspiration ranges from 0.25 to 0.70 mg/(cm^2^∙min) [17,18]. Such a significant difference in perspiration rates imposes varying requirements on the simulated testing conditions for textiles. Currently, commonly tested methods include the vertical wicking method, which provides an unlimited saturated liquid supply, and the water droplet diffusion method, along with the liquid moisture management test, which involves a limited liquid volume supply. These methods can be applied to evaluate the moisture absorption and wicking performance of textiles under different human perspiration scenarios. In addition, Kim [19] developed a new experimental setup that comprises a needle that continuously supplies liquid, mimicking a single sweat gland, to closely investigate how liquid is transported in fabrics based on within-a-yarn and yarn-to-yarn transfer wicking. Imaging techniques, such as X-rays, radiography, and tomography [20], thermocouples [21], and Neutron radiation projection and tomography [22], play a crucial role in understanding the wetting and wicking behavior, as well as the mechanisms of textiles.

The Lucas–Washburn [23,24] equation was adopted to describe the wicking dynamics of textiles. The resulting Washburn Equation (1) shows a linear functional relationship between vertical wicking height and the square root of time.

(1) $$h=\sqrt{\frac{R\gamma cos\theta }{2\eta }}t^{\frac{1}{2}}$$

where h is the wicking height, R is the equivalent radius of the capillary space, γ is the liquid-vapor surface tension, θ is the contact angle of the solid-liquid system, η is the liquid viscosity, and t is time. The slope of the line, referred to as the wicking coefficient WC, is given by Equation (2) and is determined by fitting the experimental data to Equation (1) [4]:

(2) $$WC=\sqrt{\frac{R\gamma cos\theta }{2\eta }}$$

Yarns contain complex pore and capillary channel structures with varying capillary radii at the microscopic scale, which partly limit the prediction of liquid moisture transport within textiles. Parada [25,26] reported that wicking transport occurs primarily within the yarn’s pore space formed between hydrophilic fibers. Kim [19,27] found that transfer of liquid from one capillary channel to another occurred only at contact points between the yarns and not through the space between yarns. Robert [28] presented a semi-empirical pore network model to show that pore-to-pore transition waiting times and the pore network structure affected the wicking dynamics in yarns. Most studies explain the liquid transport mechanisms within yarns under a single moisture supply condition. However, human sweat rates vary significantly under different physiological states. It is essential to investigate the liquid transport mechanisms in fabrics under varying moisture supply levels, which provide a better understanding of moisture transport behavior in textiles under different sweating conditions.

Herein, two types of twisted filament/spun combination yarns with alternating hydrophilic and hydrophobic regions along the axial direction of the yarn were developed to enhance the moisture management and comfort of textiles. The plain knitted fabrics made from these yarns were subjected to both infinite saturated liquid supply and limited droplet supply tests to simulate the moisture diffusion and liquid transport behaviors in yarns under different perspiration levels, thereby investigating the liquid transport mechanisms within the yarns inside the fabrics. This work contributes to a better understanding of the application of combination yarn in wet comfort functional textiles, as well as providing some references for testing the internal moisture wicking performance of fabrics under different human perspiration states.

## 2. Materials and Methods

### 2.1. Preparation of Twisted Profiled Filament/Spun Combination Yarn and Fabric

**Figure 1:**
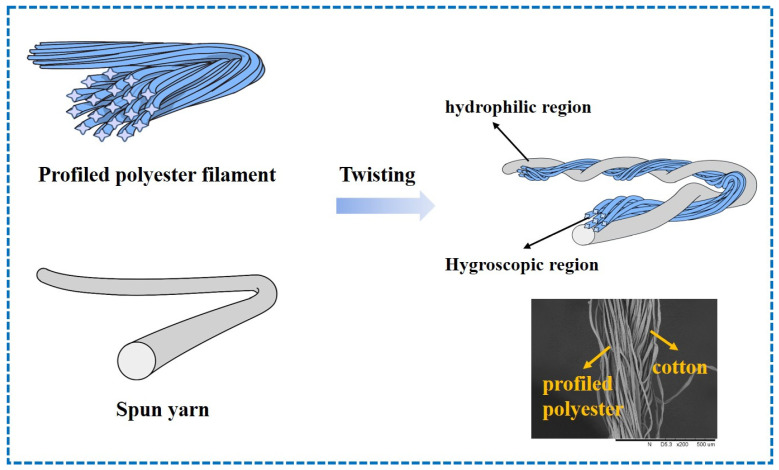
The design diagram of twisted profile filament/staple combination yarn.

The profiled polyester filament with a linear density of 75D/72F and the spun yarn with a linear density of 9.84 tex were twisted on a doubling and twisting machine to form a new type of combination yarn with a linear density of 18.45 tex and 470 twists per meter in the S direction. The design diagram of twisted profiled filament/spun combination yarn is shown in Figure 1. The spun yarns were a polyester/cotton 80/20 blended yarn and a 100% cotton yarn to form the hydrophilic region of the combination yarns, respectively. In the combination yarn, the hydrophilic single yarn (cotton yarn and cotton/polyester blend yarn), and the hydrophobic single yarn (cross-shaped cross-section polyester filament) were spirally twisted, and the hydrophilic/hydrophobic regions in the yarn are alternately spaced. A profiled polyester filament with a linear density of 150D/144F was set as a control group. The physical properties of three moisture diffusion and transport yarns are shown in Table 1. The plain knitted fabrics varying in yarn structure and composition are manufactured using a circular knitting machine, and the specifications of the fabric samples are presented in Table 2.

### 2.2. Scanning Electron Microscopy (SEM) Analysis

SEM micrographs of yarn surface topography were taken using a scanning electron microscope (TM3000, Hitachi, Tokyo, Japan). It was operating at 10 kV, 20 °C, and RH 65%. Prior to SEM evaluation, the samples were coated with a thin layer of gold by means of a plasma sputtering apparatus.

### 2.3. Yarn and Fabric Physical Properties

The properties of the yarns and fabrics were characterized according to the related standards for tensile properties of yarns (ASTM D2256M-10 [29]), yarn evenness (ASTM D1425/D1425M-14 [30]), yarn hairiness (ASTM D5647-01 [31]), fabric thickness (ISO 5084 [32]) and moisture absorption rate (ASTM D2654-89a [33]). The test specimens were conditioned for at least 24 h under standard atmospheric conditions (20 ± 2 °C, 65 ± 5% RH) and then tested in the same environment. The porosity p is calculated in accordance with Equation (3) [34]:

(3) $$p=1-\frac{V_{y}}{V_{f}}$$

where $V_{y}$ is yarn volume and $V_{f}$ is the total fabric volume.

### 2.4. Vertical Wicking Test

Wicking property of the fabrics was tested according to FZ/T 01071 [35] standard. The fabric strips (3 × 25 cm) were suspended vertically so that their lower ends were immersed in a liquid reservoir of dye solution. The distance of liquid traveling along or through a vertical fabric was recorded at specified intervals for 30 min by the scale adjacent to the samples. The test results were determined by the maximum values in the wale and course directions.

### 2.5. Droplet Diffusion Test

Liquid diffusion properties of fabrics were tested by the water drop diffusion detection device based on the image method [36]. The quantitative droplets were dropped onto a horizontally placed fabric surface, and the wetting contour images of the fabric surface were synchronously collected at a set time interval. After the polar coordinate system was established in the wetting contour image, the indices that characterized the diffusion behavior of droplets on the fabric surface were measured and calculated.

### 2.6. Moisture Management Transport Test

The property was evaluated using a Moisture Management Tester (M290, SDL Atlas, Rock Hill, SC, USA), according to the AATCC 195 [37] standard. The tested sample (8 × 8 cm^2^) was placed between the upper sensor and bottom sensor after conditioning at 65 ± 4% RH and 20 ± 2 °C to measure and record the liquid moisture transport behaviors, respectively. In this test, a total amount of water drops was set to be 200 µL and delivered for 20 s on the fabric surface. The sample was measured 5 times, and the average was taken as the final result.

## 3. Results and Discussion

### 3.1. Surface Characterization

**Figure 2:**
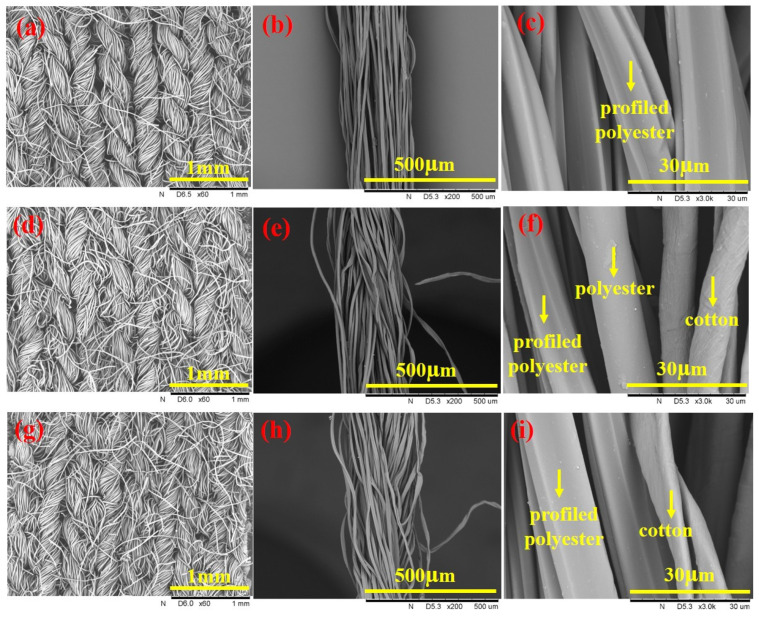
The SEM images of (**a**) S1, (**d**) S2, and (**g**) S3 knitted fabrics at ×60 magnification. (**b**,**c**) Y1 profiled polyester filament, (**e**,**f**) Y2 twisted filament/spun combination yarn, and (**h**,**i**) Y3 twisted filament/spun combination yarn at ×200 and ×3 K magnification.

Figure 2 presents SEM images of knitted fabrics, filament, and twisted combination yarns. As shown, the fabric surfaces of S2 and S3 are tighter than that of S1, which is consistent with the higher porosity of S1 compared to S2 and S3 fabrics. The profiled polyester filament Y1 exhibits smooth, continuous grooves on its surface, which can facilitate moisture conduction and diffusion along the filament. The twisted combination yarns of Y2 and Y3 contain profiled polyester filaments, round polyester staple fibers, and cotton fibers to form multiple hydrophilic-hydrophobic contact points through twisting that enhances liquid moisture transport within the yarns.

### 3.2. Comparison of Wicking

**Figure 3:**
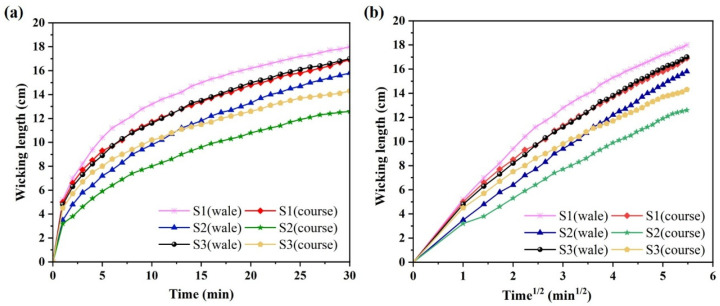
Wicking length of fabric samples (**a**) versus time and (**b**) versus the square root of time.

The wicking height can be used to evaluate the capillary transportation and moisture dissipation ability of fabrics. In the vertical wicking test, as shown in Figure 3a, the vertical wicking heights of the plain knitted fabrics formed by the three types of yarns gradually increased over time. Sample S1, formed by profiled polyester filaments, exhibited the highest wicking height along the longitudinal direction of the knitted fabric, reaching 18 cm at the end of the test. It indicates that the wicking performance of profiled polyester filaments is superior to that of twisted combination yarns, which may be attributed to the continuous capillary channels formed by the grooves on the surface of the profiled polyester filaments. In contrast, the twisted combination yarns contain hydrophilic staple fibers, and the presence of hydrophilic groups on the fiber surface has a high affinity toward water molecules in capillaries, inhibiting the liquid transfer along the capillary channels [38].

Additionally, the moisture transfer performance in the wale direction is better than that in the course direction, which may be due to liquid transfer from one capillary channel to another occurring only at contact points between the yarns and not through the space between yarns [19,27]. Knitted fabrics are formed by interlocking loops in the wale direction that, once the first loop is wetted, moisture is transferred to the adjacent loop through their contact points. Liquid transport along the tortuous path of loops in the course direction increases the transfer distance, causing a certain delay in moisture transfer. Moreover, the stitch density in the wale direction is higher than that in the course direction for fabrics, which enhances the wale wicking performance compared to the course wicking performance.

**Figure 4:**
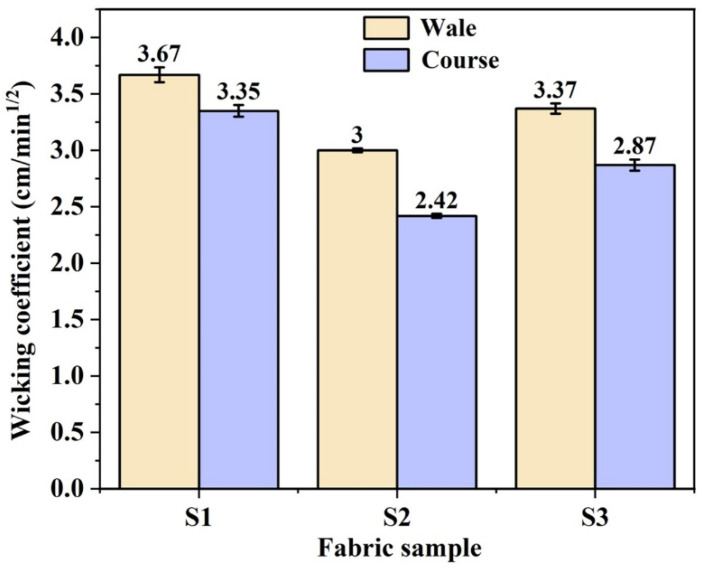
Wicking coefficients of fabric samples in both wale and course wicking directions.

The Lucas–Washburn equation, which describes liquid rise through capillary pressure in porous media, assumes an incompressible Newton fluid with infinite saturation supply and that all capillary channels have the same radius. As depicted in Figure 3b, the wicking performance of all three knitted fabrics demonstrates good agreement with the Lucas–Washburn equation, meaning its effectiveness in predicting the liquid transport property of yarns. During the initial wicking stage, samples S1 and S3 exhibited nearly identical wicking heights, indicating that both the continuous capillary channels in the yarn and the excellent moisture absorption properties could effectively enhance moisture transport performance. However, the better hydrophilicity of the twisted combination yarns caused more liquid retention within the yarn structure. This phenomenon, combined with the effects of gravitational force and viscous friction, ultimately led to a lower wicking height compared to sample S1, composed of profiled polyester filaments. Furthermore, sample S2 demonstrated the poorest wicking performance under infinite liquid supply test conditions on account of its discontinuous capillary channels, which were caused by polyester staple fibers and insufficient content of hydrophilic cotton fibers in its composition. Figure 4 presents the wicking coefficients of different fabrics in both wale and course directions. The sample S1 showed minimal difference between its wale and course wicking coefficients, whereas samples S2 and S3 exhibited significant directional variation. It was indicated that the knitted fabrics formed with twisted combination yarns demonstrated preferential moisture transport along the wale direction.

**Figure 5:**
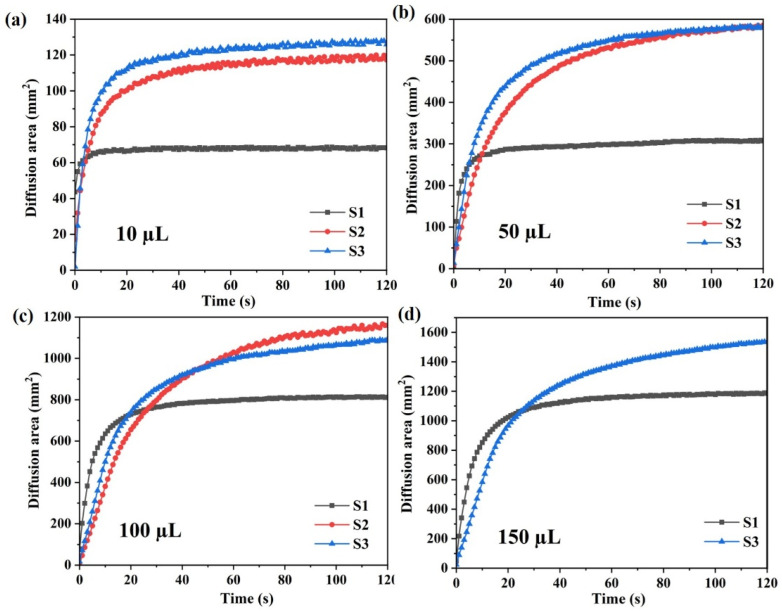
Limited droplet diffusion behaviors of samples: (**a**) 10 µL; (**b**) 50 µL; (**c**) 100 µL; (**d**) 150 µL.

In the droplet test, liquid with 150 µL is dripped onto sample S2. Due to its low internal moisture absorption rate and discontinuous capillary channels, the liquid directly penetrates the fabric surface and drips under the force of gravity, making it impossible to obtain the corresponding diffusion area data. In Figure 5a–c, it can be observed that the initial moisture diffusion rates of the three fabrics follow the order S1 > S3 > S2. Similarly, in Figure 5d, the initial moisture diffusion rate of S1 is also faster than that of S3, which is consistent with their moisture diffusion rates under infinite liquid supply conditions (vertical wicking test). It indicates that the continuous capillary channels within the fabric are more conducive to moisture diffusion and transmission in the early stages of testing. However, the hydrophilicity of the yarns in the fabric has a significant effect on moisture migration during the diffusion of droplets. Specifically, the internal moisture diffusion areas of fabrics composed of twisted combination yarns become larger than those of the profiled polyester filament fabric.

In sample S1, continuous moisture-wicking grooves in the profiled polyester filaments allow the droplet to travel along capillary channels and provide a sufficient space to retain a certain amount of liquid. For the samples of S2 and S3, due to the alternating hydrophilic and hydrophobic regions on the twisted combination yarns, the droplet was partly absorbed by the hydrophilic cotton fibers, while the rest migrated along their internal grooves in hydrophobic polyester filaments. At the contact points between the hydrophilic and hydrophobic regions, some of the liquid was transferred from the hydrophobic polyester filaments to the hydrophilic cotton fibers, while the rest continued to move under the capillary force of the yarn until all the liquid was eventually absorbed by the cotton fibers. No excess moisture remains in the grooves of the hydrophobic polyester filaments, maximizing the overall moisture diffusion area within the fabric. As a result, fabrics formed from twisted filament/spun combination yarns exhibit significantly superior moisture diffusion and wicking performance compared to the fabric made of profiled polyester filaments.

**Figure 6:**
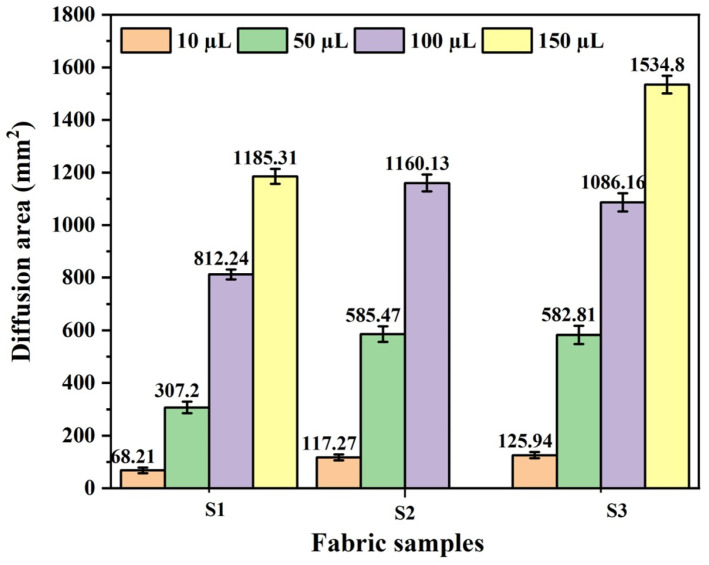
Liquid diffusion areas of knitted fabric samples.

As observed in Figure 6, the moisture diffusion area of S2 fabric is smaller than that of S3 fabric in the droplet test with 10 µL, slightly larger at 50 µL, and significantly greater at 100 µL until complete penetration occurs at 150 µL. The S2 and S3 fabrics formed from twisted combination yarns have the same porosity. However, the S3 fabric contains a higher proportion of hydrophilic cotton fibers than the S2 fabric, resulting in a higher moisture absorption rate for S3. Figure 5a shows that the initial spreading speeds of the S2 and S3 fabrics are almost the same when 10 µL of liquid is dropped, indicating that the relatively low droplet supply made the moisture tend to wick and diffuse rather than being retained by the cotton fibers in yarns. Therefore, the S3 with a higher moisture absorption rate was more conducive to moisture diffusion and wicking. Figure 5b,c reveal that the initial spreading speed of the S3 fabric is faster than that of the S2 fabric when 50 µL and 100 µL of liquid is dropped, demonstrating that its initial wicking and diffusion performance is better than that of S2. When the liquid droplet supply increases, the cotton fibers adsorb water until they reach saturation before spreading and diffusing to adjacent fibers. As a result, S2 fabric with relatively lower but more widely distributed cotton fiber content demonstrated better moisture transfer capability. However, when the liquid volume exceeds the maximum gravity that the S2 fabric can withstand, it is no longer able to transfer liquid through the fabric surface.

**Figure 7:**
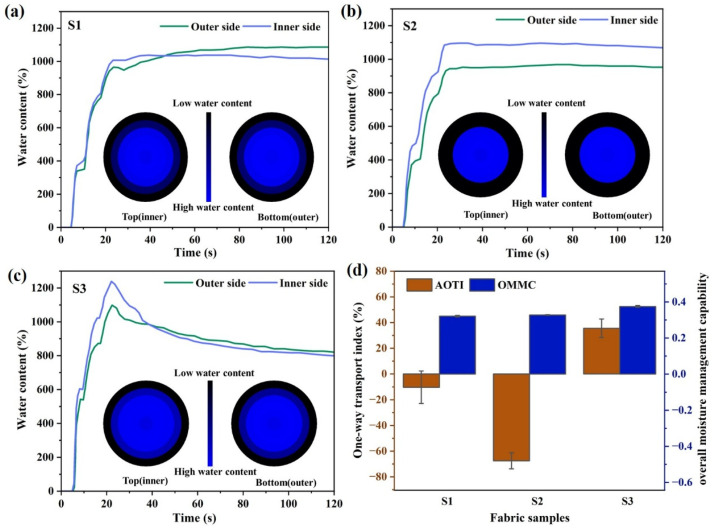
MMT results for fabric samples: (**a**) S1 fabric; (**b**) S2 fabric; (**c**) S3 fabric; (**d**) accumulative one-way transport index (AOTI) and overall moisture management capacity (OMMC) of the fabric samples.

In the moisture management test, water injection was stopped after 20 s with a total injection volume of 200 µL. In the MMT test, water injection was stopped after 20 s with a total injection volume of 200 µL. As shown in Figure 7a–c, the water content of S2 and S3 fabrics made of twisted combination yarns was higher than that of the S1 fabric composed of profiled polyester filaments. Sample S3 exhibited the highest moisture content, which was due to the relatively higher content of cotton fibers within its yarn structure. Meanwhile, S3 demonstrated the largest maximum wetting radius, indicating superior spreading and wetting properties. The inner side water content of S2 consistently remained higher than that of the outer side, and in its accumulative one-way transport index (AOTI) was the lowest in Figure 7d, suggesting that liquid moisture could not easily transfer from the skin-contact inner side to the outer side. This phenomenon may be attributed to the insufficient cotton fiber content within the twisted combination yarn, which limits the capillary-driven wetting and spreading of liquid water. Meanwhile, the polyester staple fibers fail to form continuous capillary wicking channels, resulting in partial liquid accumulation within the yarn’s internal voids. The overall moisture management capacity (OMMC) represents the comprehensive performance of dynamic liquid moisture transfer in fabrics. Sample S3 achieved the highest OMMC value, demonstrating optimal moisture absorption and one-way water transport capabilities.

From the droplet diffusion tests and MMT results of the three fabrics, it can be observed that when a limited volume of liquid water is applied to the fabric surface, internal moisture diffusion and transmission are related to the liquid volume, the content, and distribution of hydrophilic fibers. In the twisted combination yarn, the continuous capillary channels formed by profiled filaments facilitate liquid transport through capillary force, and an optimal content of hydrophilic cotton fibers spreads the liquid via absorption and wetting. The capillary forces drive the liquid within inter-yarn voids to transfer from hydrophobic polyester fibers to hydrophilic cotton fibers at their contact points. No excess liquid is retained in the grooves of the hydrophobic polyester filaments. Meanwhile, mass conservation (i.e., the liquid volume) imposes additional constraints on liquid flow. Therefore, when a limited volume of liquid is applied, the ultimate diffusion and transfer capacity depend on the optimal content and distribution of cotton fibers. In contrast, under infinite liquid supply conditions (vertical wicking test), moisture transfer on the fabric surface is driven by capillary forces, along the continuous capillary channels of the yarns. In the twisted combination yarns, the discontinuous capillary channels of polyester staple fibers and the moisture absorption of cotton fibers relatively hinder liquid rise. Consequently, the wicking performance under infinite liquid supply shows opposite trends compared to the limited-volume droplet diffusion and MMT tests.

### 3.3. Interpretation of Wicking Behavior of Twisted Combination Yarn

**Figure 8:**
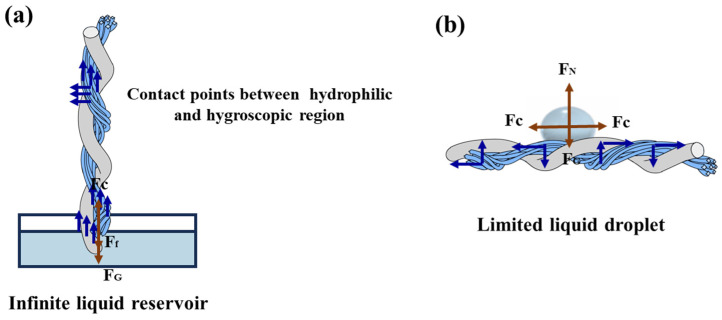
The schematic diagram of theoretical liquid water transport in the twisted profile filament/spun combination yarn: (**a**) Under an infinite liquid supply condition; (**b**) Under the limited liquid droplet supply condition.

In twisted combination yarn, liquid migrates from hydrophobic to hydrophilic regions via the hydrophilic-hydrophobic contact points since liquid transfer from one capillary channel to another occurred only at contact points between the yarns and not through the space between yarns. Under an infinite saturated liquid supply, depicted in Figure 8a, the internal liquid upward wicking is driven by the capillary force $F_{c}$ and limited by both the gravitational force $F_{G}$ and the viscous friction $F_{f}$. The balance among these three forces determined both the liquid flow velocity and the wicking height. Simultaneously, the filaments and spun yarns were in tight contact within the twisted combination yarns, forming numerous hydrophilic-hydrophobic contact points. As the liquid climbed upward under capillary force, it transferred from the hydrophobic polyester filaments to the hydrophilic spun yarns through these contact points. The higher the content of hydrophilic fibers in the spun yarns, the greater the amount of transferred moisture, resulting in increased wicking height. When a limited liquid volume was applied to the surface of twisted combination yarns (Figure 8b), the water droplet flowed internally along the yarn structure under the combined action of gravitational force $F_{G}$, supporting force $F_{N}$, and capillary driving force $F_{c}$, ultimately reaching dynamic equilibrium. Capillary forces drove the liquid within inter-yarn voids to transfer from hydrophobic polyester fibers to hydrophilic cotton fibers at their contact points, and there was no excess retained liquid in the grooves of the hydrophobic polyester filaments. However, due to mass conservation (i.e., the fixed liquid volume) imposing additional constraints on liquid movement, the yarn system exhibited optimal moisture management when satisfying the non-dripping condition. Under the constraint, the twisted combination yarns with relatively lower cotton fiber content demonstrated a larger moisture diffusion area and superior wicking capability.

## 4. Conclusions

In this paper, two types of twisted combination yarns were developed and compared with profiled polyester filament to understand the liquid migration behaviors in the plain knitted fabrics formed by these yarns. The twisted combination yarns are hydrophobic profiled polyester filament/hydrophilic pure cotton spun yarn and hydrophobic profiled polyester filament/hydrophilic polyester-cotton blended spun yarn, respectively. From the vertical wicking test method with an infinite saturated liquid supply, it was found that the hydrophobic profiled polyester filament exhibited superior liquid moisture transfer performance. The wicking behavior of all three fabrics followed the Lucas–Washburn equation, and the wicking coefficient effectively characterized the moisture transfer performance of different fabrics. Through the water droplet diffusion test with limited liquid supply and the liquid moisture management test, it was observed that the twisted combination yarns demonstrated a larger moisture diffusion area and better liquid moisture transfer performance. Different limited liquid water volumes supplied had varying effects on the internal moisture diffusion and transfer capabilities of the two types of twisted combination yarn fabrics.

The two test methods show contradictory results, indicating that the amount of liquid moisture supplied in testing conditions significantly affected the internal moisture migration performance of the twisted combination yarns during moisture transfer from the hydrophobic polyester filaments to the hydrophilic spun yarn through the contact points between the hydrophobic and hydrophilic regions. The liquid moisture in the twisted combination yarns migrated through capillary wicking for moisture transfer. Under the infinite saturated liquid supply condition, the higher the content of hydrophilic fibers in the spun yarns, the greater the amount of moisture transferred, demonstrating excellent liquid transport performance. Under the limited liquid droplet supply conditions, both the volume of liquid water and the moisture absorption capacity of the yarn jointly influence the internal moisture migration within the yarn. Fabrics formed by the twisted combination of yarns can effectively enhance moisture management performance and thermal-wet comfort for the human body during non-strenuous exercise.

## Figures and Tables

**Table 1:** Physical Properties of moisture diffusion and transport yarns.

Yarn Code	Yarn Specification	Yarn Composition	Tensile Strength /(cN/dtex)	Elongation /(%)	EvennessCV /%	Hairiness (≥3 mm)
Y1	150D/144F	Profiled polyester filament	3.24	17.35	-	-
Y2	18.45 tex	Profiled polyester filament + Polyester/cotton 80%/20%	3.15	13.33	9.57	4.6
Y3	18.45 tex	Profiled polyester filament + Cotton 100%	3.05	13.33	9.69	10.4

**Table 2:** The specification of fabric samples.

Sample Code	Yarn Composition	Porosity /%	Course Density /5 cm	Wale Density /5 cm	Fabric Thickness /mm	Mass per Square Meter /(g·m^−2^)	Moisture Absorption Rate /%
S1	Y1	64	64	96	0.61	124	0.27
S2	Y2	59	64	100	0.70	132	0.85
S3	Y3	59	64	99	0.69	130	2.69

## Data Availability

The original contributions presented in this study are included in the article. Further inquiries can be directed to the corresponding author.
